# Pregnancy in fanconi anaemia with bone marrow failure: a case report and review of the literature

**DOI:** 10.1186/s12884-017-1236-5

**Published:** 2017-02-03

**Authors:** Flavia Sorbi, Federico Mecacci, Alessandro Di Filippo, Massimiliano Fambrini

**Affiliations:** 1 Department of Biomedical, Experimental and Clinical Sciences, Division of Obstetrics and Gynecology, University of Florence, Careggi University Hospital, Viale Morgagni 85, 50134 Florence, Italy; 2 Department of Health Sciences - Division of Anaesthesiology, University of Florence, Careggi University Hospital, Florence, Italy

**Keywords:** Fanconi Anaemia, Antenatal care, High-risk pregnancy

## Abstract

**Background:**

Fanconi anaemia is a rare inherited disease characterized by congenital abnormalities, progressive bone marrow failure and predisposition to malignancy. Successful pregnancies in transplanted patients have been reported. In this paper we will describe the pregnancy of a patient with Fanconi anaemia without transplantation.

**Case presentation:**

A 34-year-old nulliparous woman with Fanconi anaemia was referred to our institution. Pregnancy was complicated by progressive pancytopenia and two severe infections. C-section was performed at 36 weeks. Both infant and mother are well.

**Conclusion:**

Successful pregnancy in a Fanconi anaemia patient with bone marrow failure is possible. The mode of delivery in patients with bone marrow failure should be determined by obstetric indications. The case highlights the safe outcome of the pregnancy with strict clinical and laboratory control by a multidisciplinary team.

## Background

Fanconi anaemia (FA) is a heterogeneous genetic syndrome characterized by congenital abnormalities, bone marrow failure (BMF), with an increased cancer occurrence [1]. Hematopoietic stem cell transplant (HSCT) using an adapted attenuated conditioning regimen, is the only curative treatment in the event of BMF [1]. Published reports show that if pregnancy occurs in FA patients after HSCT, it has a favourable outcome [1]. The effect of pregnancy in non-transplanted FA patient is unknown and pregnancy outcome has never been reported. Below is the description of the first case of a pregnancy in a non-transplanted FA patient.

## Case presentation

We report a female patient initially diagnosed with FA at age 11 years. The patient did not undergo HSCT due to lack of combined donator availability. She received periodically supportive care with leucocyte depleted and irradiated packed red blood cell (PRBC) and platelet transfusions to maintain a safe blood count.

**Fig. 1:**
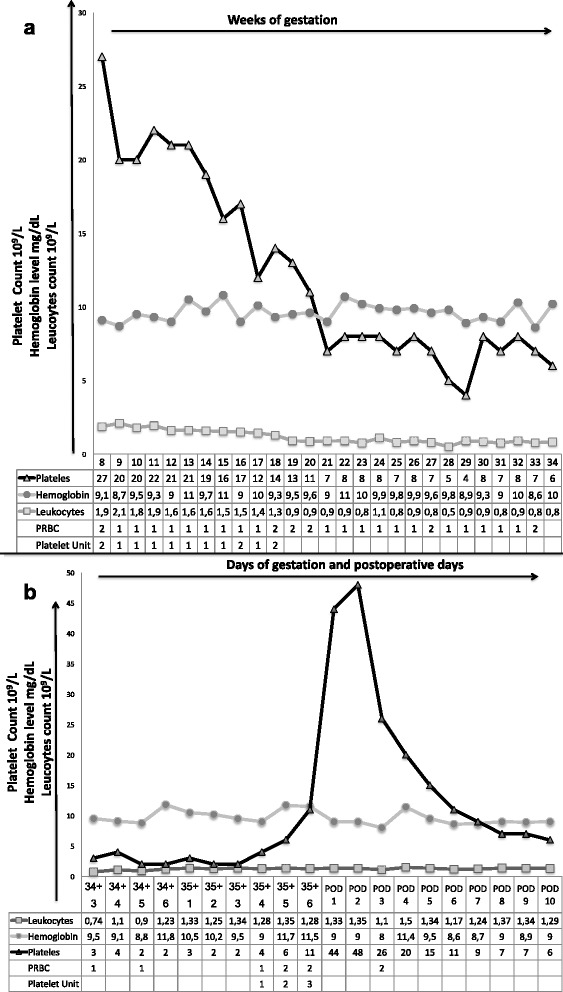
The pattern of maternal laboratory values (hemoglobin, leukocytes and platelet count) in association with component transfusion therapy (packed red blood cell and platelets) from 8 weeks of gestation to 34 weeks of gestation **a** and during patient’s last hospital stay **b** PRBC = packed red blood cells

She became pregnant naturally at 35 years old and she was referred to our institution at 8 weeks. Laboratory tests showed pancytopenia with severe neutropenia and thrombocytopenia (haemoglobin 9.1 mg/dL, leukocytes 1.86 × 10^9^/L, platelets 27 × 10^9^L). We discussed with the patient the benefits and risks of both induced abortion and continuing the pregnancy including the high probability of miscarriage, severe infections during the pregnancy, placental abruption and that pregnancy may be a precipitating factor for FA. The patient decided to continue the pregnancy. A weekly outpatient consultation was scheduled during which the pregnancy and patient’s blood tests were evaluated. Over time, pancytopenia progressed (Fig. 1a). PRBC and platelet transfusions were performed weekly to maintain haemoglobin level above of 9–10 mg/dL and platelet count above 10 × 10^9^/L in the first trimester and then of 5 × 10^9^/L.

At 15 weeks, she was admitted to our hospital with fever (102.2°F), diarrhoea and rectal bleeding. Leukocytes were 0.65 × 10^9^/L with 10% of neutrophils, platelet count was 13 × 10^9^/L, C-reactive protein (CRP) level was 180 mg/dl. Echocardiogram, chest radiography, blood and urine cultures were negative. Stool culture was positive for *Campilobacter Jejuni*. A peripheral inserted central catheter (PICC) line was inserted in order to prevent frequent venepuncture. Vancomycin 1 g every 8 h and Azithromycin 500 mg every 12 h were started. After three days of antibiotic therapy fever, diarrhoea and rectal bleeding stopped. We proposed a dose of Granulocyte colony-stimulating factor (G-CSF) but the patient refused. During hospitalization she received eight PRBC and six platelet transfusions, although survival of transfused platelets rapidly declined and she became refractor. At 20 weeks she was discharged.

At 25 weeks, she was diagnosed with gestational diabetes mellitus (GDM). We developed a customized self-monitoring of blood glucose and diet plan. Then, at 30 weeks an insulin treatment was added.

At 34 + 3 weeks she once again had high level of CRP (42 mg/dl) and was hospitalized. Blood culture was positive for *Escherichia Coli* infections. Ceftriaxone 2 g intravenous (IV) daily was started with a progressive reduction of CRP level. A caesarean section (CS) under general anaesthesia was scheduled at 35 + 6 weeks under cover infusion of three unit of PRBC and three of platelet 48 h before surgery to obtain haemoglobin level above 9 g/dL and platelet count above 5 × 10^9^/L (Fig. 1b). After ultrasound-guided placement of a jugular central venous catheter, patient was taken into the operating theatre. Three units of platelets were infused before induction, followed by two units of PRBC. Anaesthesia was induced with Propofol IV 150 mg, Remifentanil IV 0.25 μg/kg/min and Rocuronium IV 100 mg. Thereafter, anaesthesia was maintained with 0.22 μg/kg/min of Remifentanil IV. A male infant weighing 2,740 g was delivered. The baby was intubated and transferred to the neonatal intensive care unit. The Apgar scores for the baby were 3 and 7 at 1 and 5 min respectively. Umbilical cord arterial pH was 7.26. The baby was extubated after two hours uneventfully. Patient maintained stable hemodynamics intraoperatively and total blood loss was 400 ml. A bolus of ten international unit (IU) oxytocin followed by a 40 IU oxytocin infusion was administrated. At the end of procedure neuromuscular blockade were reversed with Sugammadex 500 mg IV. Following surgery, the patient was shifted to Post-operative Anaesthesia Care Unit (PACU). Antibiotics were continued with Augmentin IV 2.2 g 12 h. Enoxaparin 4000 international unit daily was started after 12 h from CS. Two units of PRBC and two units of platelets were transfused. On the second post-operative day (POD) patient was released from PACU. She once again developed a fever (100°F). Blood cultures were positive for *Staphylococcus hominis*. Meropenem 1 g IV every 8 h and Vancomycin every 12 h were started. On the sixth POD she was apyretic and was discharged on day ten.

## Discussion and conclusion﻿s

The natural history of FA ends in lethal BMF. HSCT, using an adapted attenuated conditioning regimen, is the only curative treatment when BMF occurs. In the last decade, the conditioning has been reduced because of the toxic effect of alkylating agents, making a normal ovarian function recovery and a viable pregnancy possible [1]. Our patient did not receive HSCT and decided to continue her pregnancy, although she had already developed BMF.

To our knowledge, there is no published data in relation to pregnancy in women with FA without HSCT. We relied on studies on women with aplastic anaemia (AA). AA is a pancytopenia associated with unexplained hypocellularity of the bone marrow. In literature there are many reports on pregnancy with AA, and this disorder is quite similar to our patient’s [2–4]. Data shows that patients presenting severe AA in early pregnancy should be offered termination because this may be followed by haematological improvement and because the poor maternal and fetal outcomes. Pregnant patients with thrombocytopenia are at high risk of miscarriages, placental abruption preeclampsia/eclampsia, preterm delivery, intrauterine growth restriction, fetal death, neonatal and maternal death [3]. Two studies on AA in pregnancy reported that platelet count is the main risk factor for obstetrics and desease complications [3, 4]. Most authors agree that 50 × 10^9^/L is the cut-off for therapy in asymptomatic patients [5]. Treatment is also required in patients with counts greater than 50 × 10^9^/L and bleeding complications as well as conditions associated with impaired haemostasis, such as fever [5]. Our patient began her pregnancy with a platelet count of 27 × 10^9^/L and thrombocytopenia was aggravated during pregnancy. She received weekly platelet transfusions, although from the second trimester she became refractory. Refractoriness to platelet transfusion is a common condition in patients receiving frequent platelet transfusions. An on-going process of transfused platelet destruction has been reported in severe thrombocytopenia [5]. Therefore, we decided to perform platelet transfusions only with platelet count less then 5 × 10^9^/L or with bleeding and/or fever. Moreover, due to bleeding tendency of the patient, we adopted prophylactic strategies such as a personalized self-monitoring of blood glucose scheme for the management of GDM. Intramuscular administrations were avoided.

Turning to neutropenia, this condition is defined as peripheral blood absolute neutrophil counts (ANC) below 1.5 × 10^9^/L. Authors reported an increase in neutrophil counts during pregnancy in patients with AA [3, 4]. This may explain why sepsis is a rare complication in these patients. Our patient developed a severe form of neutropenia (ANC below 0,5 × 10^9^/L) in the second trimester reaching a nadir of 0.06 × 10^9^/L at 15 weeks and then the neutrophil count partially recovered (Fig. 1). G-CSF has been shown to improve the neutropenia of patients with BMF. A recent retrospective study on 38 pregnancies with severe neutropenia demonstrated that the use of G-CSF throughout pregnancy was safe and well tolerated [6]. Our patient developed intestinal infection in the second trimester, a lower urinary tract infection at 35 weeks and a positive blood culture in postpartum. The patient refused G-CSF treatment. Even if infections were successfully managed with antibiotics, we believe G-CSF therapy could have been useful.

The patient also had severe anaemia and she received weekly PRBC transfusions. An association between severe anaemia (haemoglobin < 9 g/dL) and poor pregnancy outcome has been reported by multiple observational studies. A recent metanalysis of 44 cohort studies showed a significantly higher risk of low birth weight and preterm birth in patients with anaemia in the first or second trimester [7]. Our patient did not develop either fetal growth restriction or preterm labour, most likely due to the fact that her haemoglobin level remained above 9 mg/dl during pregnancy (Fig 1a).

This case was also an anaesthesiology challenge. As patients with thrombocytopenia should avoid invasive procedures such as venepuncture, a PICC was inserted during her first hospital stay [8]. It was used for intravenous fluids, antibiotics and to get blood samples; no complications occurred. This venous device was chosen as it has low incidence of infection and because of its low risk insertion procedure [8]. General anaesthesia was preferred over regional anaesthesia as there was a risk of spinal hematoma, due to severe thrombocytopenia. Furthermore, it would have been easier to manage severe blood loss if patient was under general anaesthesia and well oxygenated.

Concerning the mode of delivery, several investigations have stated that in patients with thrombocytopenia the mode of delivery should be determined by obstetric indications. Although some authors have concern about the risk of spontaneous intracranial haemorrhage in AA patient during labour, vaginal delivery should be the preferred route of delivery since it involves less loss of blood, as well as less risk of infections [3]. We decided to perform CS for several reasons. First, the risk of sepsis outweighed the benefit of continuing the pregnancy and a preterm delivery was necessary. Second, the patient was a preterm primigravida and induction of labour would have been unlikely successful. Finally, the date of CS was arranged two days before in order to have availability of an adequate supplies of blood products.

In conclusion, improvement in supportive treatment of FA with BMF has prolonged survival and the outlook for women to reach childbearing years is far more optimistic, than it once was. This report shows that in this high-risk patient, despite the occurrence of severe complications, pregnancy was possible without either maternal or fetal sequela. The pregnancy of our patient was managed by a multidisciplinary team (gynaecologists, haematologists and anaesthesiologists). Thrombocytopenia and neutropenia were the most challenging issues. Supportive therapy consisted of a total of 42 units of PRBC and 22 units of platelet as well as precautions against infection. We believe hereinafter information on pregnancy and disease outcome could be more accurate in non-transplanted FA patient counseling due to our experience.

### Consent

Written informed consent was obtained from the patient for publication of this Case report and any accompanying images. A copy of the written consent is available for review by the Editor of this journal.

## Abbreviations

AA: Aplastic anaemia
ANC: Absolute neutrophil counts
BMF: Bone marrow failure
CRP: C-reactive protein
CS: Caesarean section
FA: Fanconi anaemia
G-CSF: Granulocyte colony-stimulating factor
GDM: Gestational diabetes mellitus
HSCT: Hematopoietic stem cell transplant
IU: International unit
IV: Intravenous
PACU: Post-operative anaesthesia care unit
PICC: Peripheral inserted central catheter
POD: Post-operative day
PRBC: Packed red blood cell
